# The Learning Styles Neuromyth Is Still Thriving in Medical Education

**DOI:** 10.3389/fnhum.2021.708540

**Published:** 2021-08-11

**Authors:** Philip M. Newton, Hannah Farukh Najabat-Lattif, Gabriella Santiago, Atharva Salvi

**Affiliations:** Swansea University Medical School, Swansea University, Swansea, United Kingdom

**Keywords:** evidence based education, neuromyth, VARK learning style, Kolb, medical education

## Abstract

Learning Styles theory promises improved academic performance based on the identification of a personal, sensory preference for informational processing. This promise is not supported by evidence, and is in contrast to our current understanding of the neuroscience of learning. Despite this lack of evidence, prior research shows that that belief in the Learning Styles “neuromyth” remains high amongst educators of all levels, around the world. This perspective article is a follow up on prior research aimed at understanding why belief in the neuromyth of Learning Styles remains so high. We evaluated current research papers from the field of health professions education, to characterize the perspective that an educator would be given, should they search for evidence on Learning Styles. As in earlier research on Higher Education, we found that the use of Learning Style frameworks persist in education research for the health professions; 91% of 112 recent research papers published on Learning Styles are based upon the premise that Learning Styles are a useful approach to education. This is in sharp contrast to the fundamental principle of evidence-based practice within these professions. Thus any educator who sought out the research evidence on Learning Styles would be given a consistent but inaccurate endorsement of the value of a teaching technique that is not evidence based, possibly then propagating the belief in Learning Styles. Here we offer perspectives from both research and student about this apparent mismatch between educational practice and clinical practice, along with recommendations and considerations for the future.

## Introduction

In educational theory, an individual’s Learning Style is normally identified via a questionnaire which asks learners about their preferences for the way they learn, often using terms and theories that give the impression of being derived from the neuroscience of cognition (Coffield et al., 2004). Up to 70 different instruments are used in this way (Coffield et al., 2004). Amongst the most common are the VARK (Visual, Auditory, Read/Write, Kinesthetic) classification, along with Kolb’s Learning Styles Inventory and a similar system developed by Honey and Mumford (Newton, 2015). Upon identification of a preferred style, one interpretation of the theory is then that learners will achieve more if they are taught, and study, using their preferred style. This hypothesis, known as the Meshing or Matching hypothesis (Pashler et al., 2008) has been tested repeatedly and shown *not* to result in improved learning (Krätzig and Arbuthnott, 2006; Massa and Mayer, 2006; Pashler et al., 2008; Papanagnou et al., 2016; Aslaksen and Lorås, 2019; Rogowsky et al., 2020), and the reliability of the underlying preferences is often weak (Coffield et al., 2004). This misapplication of the neuroscience of learning to education has led to Learning Styles being portrayed as a “neuromyth” (Dekker et al., 2012). Belief in neuromyths has been extensively studied. Findings from our recent systematic review suggested that ∼89% of educators believe that matching instruction to Learning Styles will result in improved instruction, although there some methodological concerns about the studies reviewed (Newton and Salvi, 2020).

There is much we do know about the neuroscience of learning that could and should be applied to medical education. We know that human working memory is very limited, and that this represents a bottleneck for learning which can be managed via the techniques used in Cognitive Load Theory (Young et al., 2014). We know that the use of practice tests and other strategies that promote retrieval from long-term memory are very effective when studying clinically related topics (Dobson et al., 2017, 2018) and their use is associated with improved performance on clinical licensing exams (Deng et al., 2015). Unfortunately there is often a disconnect between good research evidence, policy and practice in Higher Education (Newton et al., 2020), and in particular, a gap between the neuroscience of learning, and educational practice (Howard-Jones, 2014).

Healthcare is a field where evidence-based practice is the gold standard (Sackett et al., 1996). It would seem reasonable to assume that the *teaching* of clinical practice would be held to a similar standard. However, a recent survey of educators showed that the most widely used teaching technique, by far, was based upon Learning Styles (Piza et al., 2019).

Thus the concept of Learning Styles appears to be an appealing one, perhaps in part due to its perceived focus on the student as an individual, even though individuals end up being lumped into 3–4 “styles.” However, healthcare training is complex. There are multiple avenues of learning required: physical dexterity, for clinical examinations and procedures; a broad understanding of multiple sciences, to be easily recalled and applied to understand complex, highly specified subjects; retention and recall of minute details of investigations and pathologies; and finally, the communication, research, compassion, empathy and diplomacy skills required for patient care. This list is by no means exhaustive. However, it does highlight one of the obvious limitations with Learning Styles theory; the mastery of these topics requires multiple sensory domains. A student who is diagnosed as an auditory learner and then tries to master dermatology using podcasts is unlikely to succeed.

One potential explanation for the persistent belief in Learning Styles is that the evidence base is itself dominated by papers which mistakenly endorse the approach, and so an educator who seeks out the “evidence” for the use of Learning Styles is given a misleading perspective. Testing this hypothesis was the basis for some of our earlier work in Higher Education (Newton, 2015), where 89% of research papers identified, about Learning Styles, in 2013–2015, mistakenly endorsed their use.

Here we repeat and extend that 2015 study, with a particular focus healthcare education. We also offer the perspective of both education research, and medical student, considering the impact of our findings on the field healthcare education as a whole.

## Methods

We followed methods used in an earlier study about Higher Education (Newton, 2015). Thus our basic research question was to characterize the picture that a Health Professions Educator would encounter were they to search the education research literature for papers about Learning Styles. As in the previous study, the inclusion criteria and analysis questions were initially applied to the abstract. If they could not be answered from the abstract, then the full text was consulted. Full text was only assessed where freely available via PubMed Central, ERIC or Google Scholar; if a subscription or payment was required, then the result was not included because access to them would vary considerably between individual health professions educators.

Two major databases were used to identify research papers; PubMed, a database focused on biomedical and life sciences, and Education Resources Information Centre (ERIC), focused on education research and information.

The term “learning styles” was the only search term used for both databases. The search was undertaken in September 2020.

### Inclusion Criteria

1. Published in the English language.
2. Published after July 2015, so as to avoid overlap with the previous study.
3. Study population from healthcare professions, e.g., medical students or qualified professionals. This included disciplines such as anatomy, pharmacy, dental, and veterinary. Review papers about health professions education were included.
4. Paper included reference, within the text of the paper, to a defined Learning Styles instrument, as listed in Coffield et al. (2004), or obviously derived from one of these instruments (e.g., the “Paragon Learning Styles Instrument” derived from the Myers-Briggs Type Indicator; Yielder et al., 2021). We did not include papers that were about “styles of learning” or other forms of personalized learning.
5. The following three analysis questions could be answered as a yes or a no.
  (a) *Did the study begin with positive intent?* Would a health professions educator be more likely than not to conclude that a premise of the study was that the use of a learning styles instrument was a useful educational approach. This could be explicit or implicit.
  (b) *Did the study end with a positive view of learning styles?* Would a health professions educator be more likely than not to conclude, having read the study, that the use of a learning styles instrument was a useful educational approach. This could be explicit or implicit. Thus studies which tested (for example) a relationship between academic achievement and Learning Styles, and found no relationship, but then advocated for further research on the topic, would be considered to have a positive outcome.
  (c) *Did the study test the “matching hypothesis”?* The matching (or meshing) hypothesis states that matching instructional activities to a supposed Learning Style will improve outcomes for individual students. This has been tested repeatedly and been shown not to work as cited earlier. Here we determined whether any studies also tested the matching hypothesis, and if so whether the results contradicted the established findings cited above that matching does not result in improved educational outcomes.

One important difference between the present study and the 2015 study was that included studies did not have to be explicitly *about* Learning Styles, just that the study had to name a specific Learning Styles instrument from Coffield et al. (2004). This change was made to test the research question more fully; a paper which endorses and encourages (or not) the use of Learning Styles will still perpetuate the myth even if it is not specifically about Learning Styles, for example papers which are testing an educational intervention and ask participants to complete a Learning Styles questionnaire as part of the evaluation.

We also identified the specific study population, country of origin and Learning Style framework used. All data were extracted by a minimum of two assessors. Any disagreement was resolved through discussion.

## Results

The initial search returned 337 results. After eliminating duplicates and studies that were included in Newton (2015), 308 results remained. Of these, 112 met the inclusion criteria for analysis. Of note was that only 10 papers were excluded for being behind a Paywall, suggesting that the bulk of the Learning Styles literature is freely available and thus there would be little incentive for a casual reader to pursue paywalled research.

### Positive Intent

109/112 (97%) of the papers started with a positive intent toward Learning Styles, i.e., a health professions educator reading the paper would, on balance, conclude that the authors initiated the study with a view that to use a Learning Styles instrument was a useful thing to do.

### Positive Outcome

102/112 (91%) of the papers concluded with a positive intent toward Learning Styles, i.e., a health professions educator would, on balance having read the paper, conclude that to use a Learning Styles instrument was a useful thing to do.

### Did the Study Test, and If So Contradict, the Meshing Hypothesis?

Only one study (Papanagnou et al., 2016) tested the Meshing Hypothesis using a recognized Learning Styles instrument. This study found no evidence to support the Meshing Hypothesis.

The most common Learning Styles instruments were the VARK system or variants thereof (e.g., VAK) (40/112, 36% of papers) and Kolb Learning Styles Inventory (35/112, 31%). Students were the most common study population, in particular Medical (36/112, 32%) and Nursing (17/112, 15%) students. The papers were from all over the world, but the United States was the most common study site (26/112, 23%).

## Discussion—Student Perspective (HNL)

As a medical student, the attraction of learning style frameworks are abundantly clear. Whilst my voice may at times appear discerning, I have personally—and multiple times—resorted to varying learning style quizzes and frameworks, seeking illumination and higher decile rankings in the form of colorful infographics… Ones often paired with promises of maps to academic success being a paywall of “only $70!” away.

Whilst amusing to reflect on, the reality of such instances is that they are borne of anxiety; paired, more often than not, with an uncomfortable need for academic validation which learning styles can offer in easy abundance. The personal preference for not wanting to run on a treadmill whilst reading from a textbook suddenly becomes proof of not being a “kinesthetic learner”; active listening becomes an auditory learning style. Clouded judgment at the hands of stress, anxiety and an overwhelming study load are waived away by the promise of a definitive answer, one that we, as medical and healthcare students, are taught to seek. In a field where such a vast body of information is required to be approached, digested and mentally filed at breakneck speed, such personalized, definitive answers may easily appear as a welcome relief.

The notion that such an innate aspect of the approach to clinical teaching is poorly evidenced is shocking, even bordering the hurtful and alarming. This is particularly true within a profession taught to rely so heavily on peer-reviewed evidence and learning.

Establishing the extent of this myth and responding accordingly is vital not only to medical and healthcare students’ wellbeing, but also the future of careers—including teaching—of many. To consider that the entire basis of our education is not as thoroughly examined as the curriculum itself, feels like a failure; and in a world of increasing fake news and hostility toward scientific evidence, seems irresponsible to perpetuate.

## General Discussion

Our findings demonstrate that an educator who was interested in understanding the evidence base for Learning Styles in Health Professions Education, and thus searched for relevant research literature, would be presented with a very misleading picture with 91% of papers presenting a positive view of the use of Learning Styles instruments.

This picture is compounded by studies in respectable journals that appeared to use experimental designs, finding significant results, but without directly testing the matching hypothesis. For example, Micheel et al. (2017) undertook a trial to test the effect of modifying an existing learning resource into multiple revised versions which were designed to accommodate preferred Learning Styles. A control group using the existing, text-based learning resource. The group that utilized a version of the resource that matched their preferred learning style did significantly better on a knowledge post-test (*P* = 0.004). Using the data presented by the authors we were able to calculate a standardized effect size which suggested that the effect was very modest (*d* = 0.06). These sorts of findings are nevertheless persuasive; this was an experimental study, conducted using a trial methodology, showing a significant improvement when participants engage with resources that match their preferred Learning Style? However, these data fail the key criteria articulated by Pashler and co; the control resource is all text. The versions used in the intervention are multimedia presentations that appear to be much more engaging; thus any improvement seen may simply be because the revised versions are just better educational resources, independent of Learning Style. Similar findings were published by Anbarasi et al. (2015) who compared the effects of a variety of different instructional materials, matched to VAK learning styles, with a “traditional group” “taught with the routine didactic lecture using PowerPoint images without pictures, videos, or animations.”

The picture is further complicated by the apparent similarities between the terminology of Learning Styles, and the language of educational neuroscience and psychology. For example, one study proposed to test the meshing hypothesis (Lehmann and Seufert, 2020) but did not use a Learning Styles instrument as defined by Coffield et al. (2004) However, they did test learners *“preferences for auditive versus visual stimuli”* using a 12-item questionnaire previously published in the German language. They then randomly assigned participants to receive visual (text) or auditory versions of a 661-word text passage, followed by measures of comprehension and cognitive load. Visual learners appeared to perform better with visual (text) material with no effect in the auditive/auditive learners. The sample here was small (*N* = 19 for auditive, 23 for visual, then split into two groups for analysis) and there is a risk of both type-1 and type-2 error (e.g., the auditory material appears to be more difficult to comprehend for all learners according to the cognitive load scores). Differential preference for, specifically, visual versus verbal content does seem to be supported by evidence, in a literature that refers specifically to cognitive “style” (Mayer and Massa, 2003), although it does not appear to impact learning achievement (Massa and Mayer, 2006).

However, the vast majority of studies did not actually test the efficacy of Learning Styles, they were instead based upon an assumption that the use of Learning Styles was a good thing. For example, a common approach was for researchers to use a Learning Styles instrument with a particular group of students studying a particular topic, and then make recommendations for changes to the teaching of that topic based upon the results.

What could, or should, be done about the persistence of this neuromyth, in a discipline for which evidence-based practice is the gold standard? A recent survey study of health professions educators found that Learning Styles was the most popular teaching technique, even when compared to aforementioned techniques which are obviously effective (Piza et al., 2019). The fact that future doctors, nurses, pharmacists etc. are still being taught using ineffective methods, supported by misleading research, is alarming. Telling educators that the techniques they believe in are ineffective is a painful message, and one that can backfire (Newton and Miah, 2017), but Learning Styles show no sign of going away. The very high belief in Learning Styles demonstrated by educators around the world does not appear to be declining over time (Newton and Salvi, 2020). The bias of research toward Learning Styles is similarly not declining; in 2015 we found that 89% of papers about Learning Styles presented a misleading positive view, and most of those were from medical education (Newton, 2015). Here 5 years later it is 91%, with dozens of papers still being published every year.

If you have got this far in reading our Perspective paper then it is likely that you also care about this, and care about teaching generally. Spread the word. Advocate for teacher development sessions where fellow educators are taught about effective approaches to Learning and Teaching (Newton et al., 2020), and maybe gently, constructively, kindly, steer your peers in a different direction when they propose the use of Learning Styles.

## Data Availability Statement

The original contributions presented in the study are included in the article/Supplementary Material, further inquiries can be directed to the corresponding author/s.

## Author Contributions

PN designed the study, extracted data from every manuscript, analyzed the data, and wrote the manuscript. HN-L extracted data from every manuscript and wrote initial draft of the manuscript. GS undertook database searches and undertook pilot data analysis. AS undertook pilot data analysis. All authors reviewed the manuscript.

## Conflict of Interest

The authors declare that the research was conducted in the absence of any commercial or financial relationships that could be construed as a potential conflict of interest.

## Publisher’s Note

All claims expressed in this article are solely those of the authors and do not necessarily represent those of their affiliated organizations, or those of the publisher, the editors and the reviewers. Any product that may be evaluated in this article, or claim that may be made by its manufacturer, is not guaranteed or endorsed by the publisher.

## References

[B1] Anbarasi M. Rajkumar G. Krishnakumar S. Rajendran P. Venkatesan R. Dinesh T. (2015). Learning style-based teaching harvests a superior comprehension of respiratory physiology. *Adv. Physiol. Educ.* 39 214–217. 10.1152/advan.00157.2014 26330041

[B2] Aslaksen K. Lorås H. (2019). Matching instruction with modality-specific learning style: effects on immediate recall and working memory performance. *Educ. Sci.* 9:32. 10.3390/educsci9010032

[B3] Coffield F. Moseley D. Hall E. Ecclestone K. (2004). *Learning Styles and Pedagogy In Post 16 Learning: A Systematic and Critical Review.* London: The Learning and Skills Research Centre.

[B4] Dekker S. Lee N. C. Howard-Jones P. Jolles J. (2012). Neuromyths in education: prevalence and predictors of misconceptions among teachers. *Front. Psychol.* 3:429. 10.3389/fpsyg.2012.00429 23087664 PMC3475349

[B5] Deng F. Gluckstein J. A. Larsen D. P. (2015). Student-directed retrieval practice is a predictor of medical licensing examination performance. *Perspect. Med. Educ.* 4 308–313. 10.1007/s40037-015-0220-x 26498443 PMC4673073

[B6] Dobson J. L. Perez J. Linderholm T. (2017). Distributed retrieval practice promotes superior recall of anatomy information. *Anat. Sci. Educ.* 10 339–347. 10.1002/ase.1668 27860396

[B7] Dobson J. Linderholm T. Perez J. (2018). Retrieval practice enhances the ability to evaluate complex physiology information. *Med. Educ.* 52 513–525. 10.1111/medu.13503 29388259

[B8] Howard-Jones P. A. (2014). Neuroscience and education: myths and messages. *Nat. Rev. Neurosci.* 15 817–824. 10.1038/nrn3817 25315391

[B9] Krätzig G. Arbuthnott K. (2006). Perceptual Learning Style and Learning Proficiency: A Test of the Hypothesis. *J. Educ. Psychol.* 98 238–246. 10.1037/0022-0663.98.1.238

[B10] Lehmann J. Seufert T. (2020). The interaction between text modality and the learner’s modality preference influences comprehension and cognitive load. *Front. Psychol.* 10:2820 10.3389/fpsyg.2019.02820 31998170 PMC6962246

[B11] Massa L. J. Mayer R. E. (2006). Testing the ati hypothesis: should multimedia instruction accommodate verbalizer-visualizer cognitive style? *Learning and Individual Differences* 16 321–335. 10.1016/j.lindif.2006.10.001

[B12] Mayer R. E. Massa L. J. (2003). Three facets of visual and verbal learners: cognitive ability. cognitive style, and learning preference. *J. Educ. Psychol.* 95 833–846. 10.1037/0022-0663.95.4.833

[B13] Micheel C. M. Anderson I. A. Lee P. Chen S.-C. Justiss K. Giuse N. B. (2017). Internet-based assessment of oncology health care professional learning style and optimization of materials for web-based learning: controlled trial with concealed allocation. *J. Med. Internet. Res.* 19:e265. 10.2196/jmir.7506 28743680 PMC5548983

[B14] Newton P. (2015). The learning styles myth is thriving in higher education. *Front. Psychol.* 6:1908. 10.3389/fpsyg.2015.01908 26696947 PMC4678182

[B15] Newton P. M. Salvi A. (2020). How common is belief in the learning styles neuromyth, and does it matter? A pragmatic systematic review. *Front. Educ.* 5. 10.3389/feduc.2020.602451 [Epub ahead of print].

[B16] Newton P. M. Da Silva A. Berry S. (2020). The case for pragmatic evidence-based higher education: a useful way forward? *Front. Educ.* 5. 10.3389/feduc.2020.583157 [Epub ahead of print].

[B17] Newton P. Miah M. (2017). Evidence-based higher education-is the learning styles ‘myth’ important? *Front. Psychol.* 8:444. 10.3389/fpsyg.2017.00444 28396647 PMC5366351

[B18] Papanagnou D. Serrano A. Barkley K. Chandra S. Governatori N. Piela N. (2016). Does tailoring instructional style to a medical student’s self-perceived learning style improve performance when teaching intravenous catheter placement? A randomized controlled study. *BMC Med. Educ.* 16:205. 10.1186/s12909-016-0720-3 27520578 PMC4983082

[B19] Pashler H. McDaniel M. Rohrer D. Bjork R. (2008). Learning styles: concepts and evidence. *Psychol. Sci. Public Interest* 9 105–119. 10.1111/j.1539-6053.2009.01038.x 26162104

[B20] Piza F. Kesselheim J. C. Perzhinsky J. Drowos J. Gillis R. Moscovici K. (2019). Awareness and usage of evidence-based learning strategies among health professions students and faculty. *Med. Teach.* 41 1411–1418. 10.1080/0142159X.2019.1645950 31407930

[B21] Rogowsky B. A. Calhoun B. M. Tallal P. (2020). Providing instruction based on students’ learning style preferences does not improve learning. *Front. Psychol.* 11:164. 10.3389/fpsyg.2020.00164 32116958 PMC7033468

[B22] Sackett D. L. Rosenberg W. M. C. Gray J. A. M. Haynes R. B. Richardson W. S. (1996). Evidence based medicine: what it is and what it isn’t. *BMJ* 312 71–72. 10.1136/bmj.312.7023.71 8555924 PMC2349778

[B23] Yielder J. Dungey G. Patten B. (2021). Personality preferences for radiation therapy students in New Zealand. *Radiography* 27 156–161. 10.1016/j.radi.2020.07.010 32747176

[B24] Young J. Q. Merrienboer J. V. Durning S. Cate O. T. (2014). Cognitive load theory: implications for medical education: AMEE guide no. 86. *Med. Teach.* 36 371–384. 10.3109/0142159X.2014.889290 24593808

